# Poorly Differentiated Thyroid Carcinoma: Single Centre Experience and Review of the Literature

**DOI:** 10.3390/jcm10225258

**Published:** 2021-11-12

**Authors:** Maria Irene Bellini, Marco Biffoni, Renato Patrone, Maria Carola Borcea, Maria Ludovica Costanzo, Tiziana Garritano, Rossella Melcarne, Rosa Menditto, Alessio Metere, Chiara Scorziello, Marco Summa, Luca Ventrone, Vito D’Andrea, Laura Giacomelli

**Affiliations:** 1 Azienda Ospedaliera San Camillo Forlanini, 00152 Rome, Italy; 2 Department of Surgical Sciences, Sapienza University of Rome, 00161 Rome, Italy; marco.biffoni@uniroma1.it (M.B.); mariacarola.borcea@uniroma1.it (M.C.B.); costanzomarialudovica@gmail.com (M.L.C.); Tiziana.garritano@uniroma1.it (T.G.); rossella.melcarne@uniroma1.it (R.M.); rosa.menditto@uniroma1.it (R.M.); chiara.scorziello@uniroma1.it (C.S.); marco.summa@uniroma1.it (M.S.); ventrone.1639886@studenti.uniroma1.it (L.V.); vito.dandrea@uniroma1.it (V.D.); laura.giacomelli@uniroma1.it (L.G.); 3 ICTUS, University of Naples Federico II, Via Pansini 5, 80131 Naples, Italy; dott.patrone@gmail.com; 4 General Surgery Department, Ospedale dei Castelli (O.D.C.), Via Nettunense Km 11,5, 00040 Rome, Italy; alessio.metere@uniroma1.it

**Keywords:** poorly differentiated thyroid cancer, total thyroidectomy, fine-needle aspiration cytology

## Abstract

There is controversy in the literature regarding a distinct subset of thyroid carcinoma whose histologically classification falls between well-differentiated and anaplastic carcinomas, previously identified as ‘poorly differentiated thyroid carcinoma’ (PDTC), or ‘insular carcinoma’, in view of the peculiar morphological characteristics of the cell groupings. The correct diagnosis and treatment of this entity have important prognostic and therapeutic significance. In this review, we describe the epidemiology, diagnosis, and management of PDTC and report our single centre experience to add to the limited evidence existing in the literature.

## 1. Epidemiology

Poorly differentiated thyroid carcinoma (PDTC) is a rare disease, with an intermediate biological behaviour between well-differentiated (papillary and follicular) and undifferentiated (anaplastic) carcinoma; it can arise de novo in the gland or represent the evolution of an unknown differentiated carcinoma [1]. According to published studies, only 27% of cases are diagnosed correctly before surgical intervention; about 80% of PDTC have a poorly differentiated component of ≥50%, and only 20% a lower percentage [2].

PDTC-reported incidence varies according to the geographic area considered: less than 1% of the whole thyroid cancers diagnosed in Japan [3], 2–3% in North American [4], and 15% of those diagnosed in northern Italy [5]. The frequency is higher in older age and more prevalent in women (2.1:1 female to male ratio), particularly in areas of endemic goitre [6].

In PDTC, regional lymph nodal or distant metastases are common at diagnosis, with about 70% of patients presenting with locally advanced disease and a median 5 years-survival of 50–60% [7].

## 2. Ultrasonographic Features

Ultrasound (US) scan of the neck is an essential imaging technique for the evaluation of thyroid disease [8], and it is used to guide fine-needle aspiration cytologic (FNAC) and core-needle biopsy procedures [9]. PDTC should be suspected when a circumscribed margin and an oval-to-round shape nodule of around 3–3.5 cm [7,10] are visualised, particularly if there is a concomitant irregular rich blood flow [11,12]. Notably, the so-called ‘sword sign’ is of particular importance, and it is observed only in poorly or undifferentiated thyroid carcinomas, with Colour Doppler US [13,14]. The reason for these appearances is supposed to be in relation to the abnormal circumscribed proliferation driven by genetic mutations and following atypical hyperplasia of thyroid follicular epithelial cells. This is commonly observed in immune diseases, as for example, in Hashimoto thyroiditis [15], and other goitre diseases where the abnormal lymphocytes stimulation might occur with higher frequency. Since only poorly differentiated or anaplastic carcinomas display external or internal jugular vein central invasion, Doppler US, preferrable during Valsalva manoeuvre, is vital to correctly plan the surgical strategy [16]. Microcalcifications presence could also be considered as a sign of malignancy in suspicious nodules, as they mostly represent psammoma bodies and might raise awareness of an occult ipsilateral or contralateral disease [17].

## 3. Histological Examination

Since its original description in 1983 by Japanese authors [18], the controversy surrounding the intrinsic nature of PDTC has been debated. Dr. Juan Rosai, who contributed significantly to this debate, observed that the main growth pattern was insular, therefore proposed the name of ‘insular carcinoma’ [19]. He also highlighted that this feature was already been described earlier on as a ‘proliferating goitre’ by Dr. Langhans, although such entity did not overlap with the solid, trabecular, and/or scirrhous patterns above identified by the Japanese authors. Thus, the controversy in the histological diagnosis, in addition to possible different geographical and ethnic factors are the main drivers of the ongoing debate.

From 2004, the WHO classified PDTC as a non-follicular non-papillary, thyroglobulin-producing thyroid carcinoma [20] with an intermediated behaviour between well-differentiated and anaplastic carcinoma, whose distinctive hallmarks of adverse prognosis are high mitotic index and the presence of necrosis.

In 2006, a proposed diagnostic algorithm, known as the ‘Turin proposal’ defined the following diagnostic criteria [21]: (1) presence of a solid/trabecular/insular pattern of growth in a malignant (invasive) thyroid lesion of follicular derivation in the majority of the tumour; (2) lack of the conventional papillary carcinoma nuclear features; (3) presence of mitotic activity >3 × 10 HPF or tumour necrosis or convoluted nuclei. Albeit simplifying the diagnosis, limitations inherent criteria reproducibility have been highlighted [22]—namely, what percentage of poorly differentiated tissue was needed in a tumour to allow for such a diagnosis and to affect patient prognosis, specifically considering the fact that the 2004 WHO classification did not offer a cut-off value.

Finally, the Memorial Sloan Kettering Cancer Centre (MSKCC) criteria for PDTC are less restrictive and only consider an elevated mitotic index (>5/10 HPFs) and/or tumour necrosis regardless of tumour growth patterns and nuclear aspects [23].

## 4. Histological Variants of Poorly Differentiated Carcinoma

The oncocytic variant of PDTC is even more controversial, and although it is recognised as an independent entity by the WHO [20], it was not originally included in the Turin proposal. The presence of necrosis in this tumour variant is the main characteristic, which is common to oncocytic lesions in general, where spontaneous or FNA-initiated infarction and focal necrosis often occur. It is important, however, to identify oncocytic PDTC early, as it is associated with worse outcomes in comparison with conventional PDTC [24].

## 5. Cytology: The Bethesda System for Reporting Thyroid Cytopathology (TBSRTC)

The needle aspiration under ultrasound guidance is the most frequently used examination for diagnostic purposes [25]; however, the amount of material may be low or inadequate for diagnosis. In these cases, with high suspicion of malignancy, a core biopsy is recommended rather than FNAC repetition to increase the accuracy of percutaneous needle diagnostics, as a first-line tool in selected cases [26].

The Bethesda System for Reporting Thyroid Cytopathology (TBSRTC) regulates the management of patients after FNAC [27], as summarised in Table 1.

A difference exists for category IV (follicular neoplasm) cases which undergo hemithyroidectomy and category V (suspected malignancy) which undergo total thyroidectomy. Single cases of PDTC might be placed in either of these categories because of morphological overlapping, as previously mentioned, but surgical intervention is always recommended, given the malignant nature of PDTC. Furthermore, it has been reported that only 32.5% of PDTC cases are correctly diagnosed by FNAC, whose main feature appears to be the architectural pattern of cellular nests and three-dimensional clusters, along with loosely cohesive singly dispersed cells in the background. This latter feature represents a highly distinctive tract of PDTC.

In view of the controversy related to the interpretation of the cytological appearance, an integration with ultrasound findings might be useful in the intermediate (IV) Bethesda category, where completion of the thyroidectomy would be the safest approach if an initial lobectomy was performed. However, in cases where morphological features are indicative of TBSRTC category V or VI, a preoperative histological diagnosis before surgery is not necessary, as the treatment will not be affected.

## 6. Immunohistochemistry

In addition to the cytohistological tracts, the diagnosis is based on a panel of immunohistochemical stains, summarised in Table 2. Although PDTC loses the component of well-differentiated thyroid carcinoma, it produces thyroglobulin, contains colloids, and retains the ability to respond to radioactive iodine [28]. Furthermore, since it originates from a gland, it is derived from epithelial cells, thus maintaining the immunophenotypic characteristic of expressing cytokeratins, as reported by Dettmer et al. [29].

## 7. Molecular Biology

BRAF and RAS mutations remain the principal genes involved in aggressive thyroid carcinomas, occurring in 33% and 45% of the PDTCs, respectively [30,31]. Notably, there is a correlation between the genes involved and the phenotype displayed, with 42% of RAS mutations in identifiable PDTCs according to both Turin proposal and MSKCC criteria, and with BRAF mutation only accounting for 78% of the MSKCC-diagnosed PDTCs. Additionally, BRAF-mutated PDTCs are more frequently responsible for a loco-regional disease, while on the contrary, RAS-mutated follicular carcinomas tend to present with distant metastases.

It has also been demonstrated that the co-existence of BRAF/RAS and TERT genetic alterations has a detrimental impact on the aggressiveness of thyroid carcinoma. More specifically, TERT promoter and TP53 mutations, as well as PIK3CA–PTEN–AKT–mTOR pathway, SWI–SNG complex synergistically concur to worse outcomes in PDTC [32].

The median mutation burden detected in PDTC is 2, and an above-median number of somatic mutations is associated with a larger tumour size of >4 cm, a higher frequency of distant metastasis, and shorter overall survival [30].

## 8. TNM Classification

In October 2016, the American Joint Committee on Cancer (AJCC) published the 8th edition of the AJCC/TNM cancer staging system, and it has been introduced in clinical practice since 1 January 2018 [33,34] (Table 3).

## 9. Management

As for well-differentiated thyroid cancers, the initial phase is managed by the endocrinologist and the surgeon, with an adequate staging of the disease in a short time, the main requirement to correctly plan the treatment and achieve the best recurrence-free survival outcomes [7,10]. The initial diagnosis is clinical and cytohistological. The presence of rapidly growing thyroid nodule with a tendency to involve loco-regional structures (nodes), but also to eventually metastasise, is already clinically suggestive of an aggressive carcinoma. It is recommended to consider the clinical, US, and cytological major features including the rapid growth of a well-defined mass, as well as its heterogeneity and hypoechogenicity, trying to target the strong hypoechoic area when performing the FNAC. The same alert should rise for fast-growing suspicious lymph nodes, mainly at the subcortical area (where the metastatic cell nests develop; in fact, necrosis is often acellular and located centrally within the metastatic node), to give rise to the diagnosis of PDTC.

Yet, as previously mentioned, cytological diagnosis of PDTC based on FNAC is challenging, in view of the rarity of the disease, the nonspecific cytological features, the overlap with cytological characteristics of follicular neoplasms, and the frequent presence of the poorly differentiated component within the well-differentiated tumour.

Elimination of PDTC can be achieved by complete surgical removal and treatment of a limited loco-regional disease, with a high remission (94.3%) [35], very close to that of well-differentiated carcinoma and superior to that of anaplastic carcinoma [36]. The increase in survival is associated with the young age (<60 years), the limited size of the tumour, the absence of distant metastases, the co-existence of a well-differentiated thyroid tumour, and a greater extension of neck surgery, as indicated in Table 4, our single centre experience. Surgical intervention in operable cases involves total thyroidectomy associated with complete recurrent and lateral cervical lymphadenectomy.

Our previous experience also showed that the presence of lateral cervical lymph nodes at the time of diagnosis is higher for patients older than 71 years [37], confirming the significance of age as a prognostic factor, especially in thyroid cancers.

Finally, in terms of follow-up, given the differentiation of the thyroid cell, the use of thyroglobulin dosage in the follow-up is an indicator for relapse of the disease, and for the same reason, there is support for the use of a suppressive hormone replacement therapy or for the use of radioactive iodine (even in the presence of mixed forms). This is particularly relevant in the radioiodine-resistant forms, and even though chemotherapy is currently not standard of care, emerging positive effects have been reported into two large trials [38,39], in which monoclonal antibodies, sorafenib and lenvatinib, were administered.

## 10. Conclusions

Poorly differentiated thyroid carcinoma diagnosis is challenging; thus, this disease might remain underdiagnosed. The Turin proposal—namely, a high mitotic index and a solid/trabecular or insular pattern, are the most broadly in use diagnostic algorithm, and it is important to determine the percentage of poorly differentiated disease to correctly estimate recurrence-free survival and plan treatment accordingly. The genetic landscape of PDTC is in continuous evolution and surgical radical treatment offers excellent survival.

## Figures and Tables

**Table 1:** The Bethesda System for Reporting Thyroid Cytopathology (TBSRTC) classification.

Diagnostic Category	Risk of Malignancy (%)	Recommendation
I. Non-diagnostic or unsatisfactory	1–4	Repeat FNAC with Ultrasound guidance
II. Benign	0–3	Clinical Follow-up
III. AUS or FLUS	5–15	Repeat FNAC
IV. Suspected follicular neoplasm/Follicular neoplasm	15–30	Hemithyroidectomy
V. Suspected Malignancy	60–75	Near total thyroidectomy or hemithyroidectomy
VI. Malignancy	97–99	Total thyroidectomy

AUS: Atypia of undetermined significance; FLUS: follicular lesion of undetermined significance; FNAC: fine-needle aspiration cytology.

**Table 2:** Common immunohistochemical staining used in poorly differentiated thyroid carcinoma (PDTC).

Immunohistochemical Staining	PDTC
Calcitonin	−
Chromogranin A	no data
Synaptophysin	no data
Thyroglobulin	+
Galectin-3	−/+
HBME-1	−/+
PanCK	+
TTF1	−/+
CK7	−/+
CK19	−/+
PAX8	−/+

**Table 3:** TNM classification.

Primary tumour (pT):
TX: Primary tumour cannot be assessed
T0: No evidence of primary tumour
T1: Tumour ≤2 cm in greatest dimension limited to the thyroid
T1a: Tumour ≤1 cm in greatest dimension limited to the thyroid
T1b: Tumour >1 cm but ≤2 cm in greatest dimension limited to the thyroid
T2: Tumour >2 cm but ≤4 cm in greatest dimension limited to the thyroid
T3: Tumour >4 cm limited to the thyroid or gross extrathyroidal extension invading only strap muscles
T3a: Tumour >4 cm limited to the thyroid
T3b: Gross extrathyroidal extension invading only strap muscles (sternohyoid, sternothyroid, thyrohyoid, or omohyoid muscles) from a tumour of any size
T4: Includes gross extrathyroidal extension into major neck structures
T4a: Gross extrathyroidal extension invading subcutaneous soft tissues, larynx, trachea, oesophagus, or recurrent laryngeal nerve from a tumour of any size
T4b: Gross extrathyroidal extension invading prevertebral fascia or encasing carotid artery or mediastinal vessels from a tumour of any size
Regional lymph node (pN):
NX: Regional lymph nodes cannot be assessed
N0: No evidence of regional lymph node metastasis
N0a: One or more cytologic or histologically confirmed benign lymph nodes
N0b: No radiologic or clinical evidence of locoregional lymph node metastasis
N1: Metastasis to regional nodes
N1a: Metastasis to level VI or VII (pretracheal, paratracheal, prelaryngeal/Delphian or upper mediastinal) lymph nodes; this can be unilateral or bilateral disease
N1b: Metastasis to unilateral, bilateral, or contralateral lateral neck lymph nodes (levels I, II, III, IV, or V) or retropharyngeal lymph nodes
Distant metastasis (M):
M0: No distant metastasis
M1: Distant metastasis

**Table 4:** Our single centre experience.

Pt	Sex	Age (Years)	FU (Months)	FNAC	AP (mm)	T (mm)	L (mm)	Surgery	Histology	Lymphadenectomy	pT	pN
1	M	58	60	-	-	-	-	TT	PDTC	Radical lymphadenectomy + laryngectomy	4a	0/8
2	F	85	24	4	-	-	-	TT	PDTC	Periglandular nodes	4a	1a 1/2
3	M	64	24	4	24	13	27	TT	PDTC	2 loco-regional nodes	4a	0/2
4	F	54	24	recurrence	7.5; 18.9	4.6; 8.8	11.6; 20	Nodule removal	PDTC	-	-	-
4	F	55	12	recurrence x2	0.08	0.05	0.05	Nodule removal	PDTC	-	-	-
5	M	56	12	3	46	38	41	TT	PDTC	N	3a	-
6	F	55	12	3	50.8	37.7	61.2	TT	Oncocytic	Latero-cervical II-IV	3b	1b (10/43)
7	F	80	12	-	-	-	-	TT	PDTC	Latero-cervical III-IV-VI-VIII	4	1b 6/44

AP: anteroposterior diameter; FNAC: fine-needle aspiration cytology; FU: follow-up; L: lateral diameter; pN: primary nodes; pT: primary tumour; Pt: patient; T: transversal diameter; TT: total thyroidectomy.

## Data Availability

The data used to support the findings of this study are included within the article and are available on request from the corresponding author.

## Abbreviations

FNAC	Fine-needle aspiration cytology
PDTC	Poorly differentiated thyroid carcinoma
TBSRTC	Bethesda System for Reporting Thyroid Cytopathology
US	Ultrasound
